# Supplementary material for: Are carbon emissions trading and green financial instruments synergistic? -Comprehensive quantitative research based on content analysis
Source: PLoS One. 2024 Mar 7;19(3):e0298601. doi: 10.1371/journal.pone.0298601 (PMC10919601; doi:10.1371/journal.pone.0298601)
Supplement: S1 Data — (ZIP) [file pone.0298601.s001.zip › Data/地级市Z_F工作报告.zip!/╣π╢1⁄2/╒╪╟∞╩╨/└┤╫╘╣1⁄2╓┌║┼í╛┬φ┐╦╩2╛▌═°í┐_2017.docx]

各位代表：

　　我代表肇庆市人民政府向大会作工作报告，请予审议，并请政协委员和其他列席人员提出意见。

2016年和本届政府工作回顾

　　去年是“十三五”发展的开局之年。一年来，面对持续加大的经济下行压力，在省委、省政府和市委的正确领导下，在市人大和市政协的监督支持下，市政府紧紧围绕建设珠三角连接大西南枢纽门户城市的目标，加快推进供给侧结构性改革，在抓好稳增长、调结构、惠民生的同时，着力谋长远、打基础、增后劲，推动经济社会发展取得新成效，经济实现平稳增长，城乡建设不断加快，社会事业全面进步，社会大局和谐稳定，迈出“十三五”发展坚实步伐。

　　一、经济发展稳中有进

　　全市预计（下同）完成地区生产总值2070亿元，比上年增长（以下简称“增长”）5%左右，人均生产总值50800元，增长4.2%，一、二、三产业增加值分别增长4%、3.6%、7.6%；规模以上工业增加值965亿元，增长3.6%；固定资产投资1369亿元，增长3%左右；社会消费品零售总额725亿元，增长11.8%；外贸出口总额311.5亿元，增长5%；一般公共预算收入91.69亿元，下降34.6%，剔除化解超常规收入和“营改增”、免征涉企收费政策性减收后增长1.33%，非税占比由48.9%下降到30.5%，财政收入质量全省排名从第21位提高到第6位；金融机构本外币存款余额首次突破2000亿元，增长12%；城镇和农村常住居民人均可支配收入分别为25800元、15100元，分别增长9%左右和8.5%左右。

　　供给侧结构性改革全面启动。全面实施“三去一降一补”五大行动计划。市属国有关停企业全部出清。推进租售并举住房制度改革，在全省率先启动专业化住房租赁平台试点，开展不动产统一登记，公积金贷款办理时限缩短三分之二以上，消化商品房库存37.34万平方米。打好降低企业成本“组合拳”，为企业减负49.76亿元。全面落实“营改增”扩围政策，新增试点四大行业税负只减不增，清理规范29项行政审批中介服务。

　　实体经济稳定增长。常态化开展“暖企行动”，分别安排中小微企业贷款风险补偿基金和应急转贷资金1.7亿元、2亿元，办理“过桥融资”业务2亿元，召开政银企对接合作会帮助663家企业贷款365亿元，比上年对接会增长59.7%。“规下”转“规上”工业企业47家，新增上市、新三板挂牌企业10家，新增区域股权交易市场注册挂牌企业8家。完成工业投资734亿元，总量位居全省前列，其中完成工业技改投资103.55亿元，增长46.5%，增速居全省第6位。四大工业主导产业实现增加值234亿元，增长2.5%。出台支持绿色环保产业发展政策措施，与丹麦共建环保产业园。新经济、新业态蓬勃发展，大旺跨境贸易电商平台试运行，唯品会二期等11个项目动工建设，新引进京东集团“一基地三中心”和阿里巴巴农村淘宝项目，肇庆大数据云服务产业园成为省首批大数据产业园，全市限额以上单位通过公共网络实现商品零售额89亿元，增长58%。全市景区接待游客3300万人次，实现旅游收入290亿元，分别增长12.7%、20%。现代服务业增加值占服务业比重达45.2%。

　　创新能力不断增强。全面落实珠三角（肇庆）国家自主创新示范区发展规划纲要。肇庆高新区制定实施创新创业“三年行动计划”，智能制造产业园和广东工业生物技术转移中心等项目加快建设。我市与环保部华南环境科学研究所开展环保战略合作，与暨南大学共建绿色发展研究院，肇梦空间成为国家级众创空间和省首批“互联网+小镇”培育单位，国家建筑五金产品质量监督检验中心正式运行。新增省级工程技术研究中心15家，高新技术企业数量、发明专利申请量和授权量分别增长35.3%、68.2%、19.4%，通过国家知识产权试点城市考核验收。大力实施“西江人才计划”，引进4名国家级人才，建立全国包装饮用水行业首家企业院士工作站，建成首家省市共建人才驿站，启动建设高层次人才双创园，成功举办首届创新创业大赛。

　　重点项目加快推进。100项市重点项目完成投资496.71亿元，完成年度投资计划109%，其中51项省重点项目完成投资计划128%，完成进度居全省第2位。华润水泥第六条生产线、鼎丰公司增资扩产等项目建成投产，德庆大顶山风电场发电运营，海王健康产业园等项目开工建设，高要鸿图科技园、封开绿色环保骨料等一批产业项目落户。强化融资用地等要素保障，争取各类资金呈现爆发式增长，实际有效融资余额1781亿元、比年初增长13.8%，新获批政府性投资项目融资661亿元，获得上级扶持资金324亿元，企业发行债券53亿元，分别增长6.6倍、90%和4.9倍。新获批建设用地3万亩，实际供地超过4.3万亩，盘活闲置土地3000亩，清理拆除违法建筑38万平方米。

　　二、“三大抓手”取得突破

　　市政交通设施提速建设。组织开展新一轮市政交通建设大会战，广佛肇城际轨道正式开通，广佛肇高速肇庆段提前一年多通车，汕湛、汕昆、怀阳高速肇庆段完成年度投资，肇庆火车站综合体、肇庆东站站前综合体动工建设，西江航道肇庆段扩能升级工程完成总投资55%，肇佛对接“两桥一路”（大旺至三水北江大桥、高要金利至三水白坭西江大桥、肇庆至开平高速公路）前期工作抓紧推进。大冲枢纽立交建成通车，北岭路网全线动工、一期工程基本建成，阅江大桥主桥合龙，321国道城市化道路改造一、二期工程全面铺开，江滨堤路改造升级工程正式动工。

　　园区承载能力有效提升。出台鼓励工业园区建设和使用通用厂房指导意见，新增及盘活园区开发面积8.2平方公里，建成工业厂房168.6万平方米，其中建成工业通用厂房59.8万平方米。肇庆高新区盘活用地630亩、厂房14.6万平方米，启动建设3000亩产业园中园。粤桂合作特别试验区（肇庆）基础设施投资增长80%，新增开发面积1600亩。广佛肇经济合作区起步区完成基础路网建设，开发面积增加到1.04万亩。肇庆（高要）汽车零部件产业园被纳入广州国际汽车零部件产业基地拓展区，完成首期填土工程。

　　城市扩容提质不断加快。出台市域规划一张蓝图实施方案，统筹推进“三规合一”。肇庆新区全面启动20个重点项目建设，投资81亿元的综合管廊和投资51亿元的四大水系等重点工程全面动工，完成政府性项目投资75.86亿元，新获批融资248亿元、增长6.2倍，新征收土地1.9万亩，中国优质农产品交易中心投入运营，商务中心竣工启用，中山大学附属第三医院肇庆医院和保利商务中心主体封顶，启迪环保科技城动工建设，北大医疗康复医院肇庆分院等项目加快实施。肇庆府城保护与复兴项目从谋划转入启动，已完成征收房屋922间、面积7万平方米，古城墙正在联合申报世界文化遗产。端州城区50条“瓶颈路”全部立项，22条完成征拆，人民北路等19条“瓶颈路”率先交付施工，累计完成征拆房屋977间、面积11万平方米。深入实施创文“8+1”专项整治行动，城市面貌明显改善。四会市成为第三批国家新型城镇化综合试点。高要区回龙镇成为全国首批特色小镇。

　　三、开放合作迈出新步

　　大招商招大商成效显著。成立市招商委员会和12个招商引资（智）工作小组，成功举办2016中国环保上市公司峰会暨“肇庆金秋”经贸洽谈会，签约合同项目54宗，投资总额603.6亿元。出台《招商引资鼓励暂行办法》，全年引进合同项目172宗、计划投资总额约702.7亿元，其中超10亿项目13宗、占投资总额47.5%。招大引强取得重大突破，首期投资60亿元的新能源动力小镇以及分别投资30亿元的中国电科肇庆产业园、广东中电新能源汽车、益华全球商品流通展示体验中心等重大项目签约落户，引进了恒大文化旅游城、金域集团鼎湖健康养生城等重大服务业项目。

　　区域合作共建更加紧密。积极参与“广佛肇清云韶”共建合作，与广佛共同谋划推进新一轮交通对接，积极开展总部在广佛、生产在肇庆的产业共建，深化与广佛整车基地合作。成功举办首届珠江—西江经济带沿线城市联合招商推介会暨西江绿色发展论坛，共同发表保护西江绿色发展《肇庆宣言》。与粤桂黔十二市（州）政府签订共建粤桂黔高铁经济带《行动计划》。

　　四、“三农”工作扎实推进

　　精准脱贫攻坚首战告捷。全市111条贫困村、4.2万贫困户全部落实挂钩帮扶责任，市县两级共派出1146个单位、1200多名干部进村入户。在省直单位和中山市大力支持下，累计筹集扶贫资金5.1亿元，贫困户精准识别和建档立卡全面完成，因户施策精准制定帮扶规划，落实政府兜底政策，3.2万贫困人口实现脱贫，圆满完成年度扶贫脱贫任务。

　　现代农业发展加快。农林牧渔业总产值、增加值增速继续居全省前列。国家级粮食创高产示范片建设扎实推进，粮食生产实现“八连增”。广东（高要）粤台农业合作试验区核心区动工建设。壮大发展农业经营主体，培育创建年销售收入超5亿元的大型骨干农业企业2家，新增农民专业合作社140家，新建国家级农业标准化示范区5个，新增省级农业名牌产品9个、无公害农产品8个。全面建成县级农业综合执法机构和镇（乡）农产品检测站。新建省级休闲农业与乡村旅游示范镇1个。

　　农村发展基础巩固提升。完成高标准基本农田改造21.39万亩，新建农村硬底化公路350公里，农村电网改造走在全省前列，实现广播电视“村村通”。三批省级新农村连片示范建设累计完成投资2.98亿元，新增省名村25个，德庆被确定为全国农村产业融合发展试点示范县。农村土地承包经营权确权登记颁证全面推进，累计完成实测面积143.73万亩。农村“三资”管理服务平台累计成交21.6亿元。全面完成省普惠金融“村村通”试点工作，建设信用村460个、乡村金融（保险）服务站476个。建成县镇村三级公共法律服务平台。

　　五、民生事业全面发展

　　生态环保建设强力推进。组织实施“环境执法年”活动，实施领导干部生态环境损害责任追究和环境空气质量排名公布制度，切实做到“三个绝不”。深化大气污染五大领域源头治理，城区空气质量优良天数320天，占比为87.4%，提高1.4个百分点。成立肇庆西江保护管理和综合利用委员会，严厉打击西江河道非法采（运）砂和非法砂场经营行为，实施“河（涌）长制”，“一河（涌）一策”治理7条河涌和黑臭水体，实施羚山涌截污清淤等工程，星湖水质整治二期工程基本完成，新建成污水处理厂12座，大江大河和饮用水源地水质稳定达标。开展土壤环境保护和综合治理。以中央环保督查为契机，认真抓好自查自纠和195宗交办案件办理，关停取缔企业65家，行政拘留7人，问责91人次。完成林业重点生态工程建设任务，新增森林公园22个、湿地公园2个，羚羊峡古栈道森林公园建成免费开放，绿化美化乡村176个，成功创建成为国家森林城市。

　　社会各项事业蓬勃发展。财政投入民生类支出185.3亿元，占一般公共预算支出比重达74.5%，我市承办的省民生实事任务全面完成，市十件惠民实事完成年度任务（具体内容见附件一）。发放各项就业创业补贴1.09亿元，城镇新增就业4.35万人，失业率控制在2.37%以内。启动新建中小学校4所、扩建15所，增加学位7670个，高要、四会创建成为省推进教育现代化先进区（市）。成为公立医院改革国家联系试点城市，完成16所乡镇卫生院标准化建设，建成100个公建民营规范化村卫生站。德庆县创建成为省卫生县城，新建省、市卫生村156个。完成农村危房改造3103户，开工棚户区改造6310户，新增公租房（含租赁补贴）714套（户），建成保障性住房、棚户区改造住房6370套（户），鼎湖区成为全国保障性住房“购租并举”试点和省棚改“房票”制度改革试点。实施古城墙抢修加固工程，完成高要学宫修缮，肇庆艺术中心建成投入使用，市博物馆新馆、肇庆文化发展中心加快建设。成功举办首届肇庆国际半程马拉松赛、首届鼎湖山音乐节、“美丽中国·肇庆合唱周”和广东跨年嘉年华。第十届市运会圆满成功，省运会场馆建设改造全面推进，城区新建社区体育公园10个。连续七届荣获“省双拥模范城”称号。怀集县下帅乡被命名为“第四批全国民族团结进步创建活动示范单位”。食品药品安全、安全生产、社会治安形势总体稳定。计生、统计、民族、宗教、外事、侨务、港澳、对台、人防、国防动员、地方志、档案、气象、水文、地震、妇女儿童、老龄、残疾人、慈善等工作取得新成绩。

　　六、政府建设不断加强

　　自觉接受市人大法律监督和市政协民主监督，按期办结人大代表建议124件、政协提案138件，配合市人大开展城区市容和环境卫生管理、古城墙保护、端砚保护等立法起草工作，市县两级全部建立政府法律顾问制度，启动实施“七五”普法，法治政府建设稳步推进。全面实行“一门式一网式”服务模式，企业工商登记实现“五证合一”，实施“双随机、一公开”监管，新登记企业户数和注册资本（金）分别增长31.4%、95.7%。认真开展“两学一做”学习教育。切实加强行政监察、审计监督，建成政府投资工程廉情预警评估系统，源头治腐不断深化，整治“为官不为”力度加大，积极构建新型政商关系。大力推进政务公开，自觉接受网络问政、《市民问政》等社会监督，在省政府门户网站参加“市长面对面”专访。

　　各位代表，去年全市经济社会发展取得的新成效，标志着本届政府各项任务顺利完成。五年来，我们深入落实《珠三角规划纲要》和《加快建设枢纽门户城市行动计划》，大力实施“两区引领两化”战略，推动经济结构战略性转型，经济社会实现持续较快发展，为建设成为枢纽门户城市打下坚实基础。综合实力稳步提升。地区生产总值年均增长9.1%，从1000亿跨越到2000亿。固定资产投资累计完成5699亿元，实现五年翻番。三次产业加快发展，产业结构调整为14.9:48.6:36.5。“两化”进程持续加快。工业主导地位更加突出，2000亩以上规模园区增加到22个，省级产业转移园（集聚地）实现县域全覆盖，有3个行业年产值超500亿元，工业总产值从2468亿元跃升到超4000亿元，肇庆高新区成为国家自主创新示范区。肇庆新区实现“三年打基础”，端州老城功能不断提升，高要完成撤市设区，市辖区面积和中心城区规划面积分别扩大3.1倍、2.4倍，城市东拓南连取得历史性突破。交通枢纽逐步成型。累计完成交通投资527亿元，建成和在建高速公路477.4公里，通车里程五年翻番，进入“高铁时代”和“城轨时代”，高铁通车里程居全省首位，“一条城轨、两条高铁、七条高速、一条黄金水道”的综合交通网络初步构建。改革创新力度加大。行政审批、商事制度、投融资体制等改革加快推进，行政审批服务从“一楼式”升级为“一窗式”、“一网式”，123项行政审批事项实现“零跑动”办理，政府公共服务满意度连续三年居全省前五位。投融资改革不断深化，近两年各类融资大幅增长。中央农办农村综合改革试点和国家“多规合一”、简政强镇等试点成效初显。创新驱动步伐不断加快，高新技术企业数量实现翻番，成为国家产学研合作创新示范基地、省部产学研合作示范市和首批省级小微企业创业创新基地城市示范点。开放合作全面深化。广佛肇经济圈加快建设，粤桂合作特别试验区成为我国首个东西部省际合作示范区，广佛肇经济合作区上升为省级平台，5年累计吸收外资54.9亿美元，累计承接产业项目412个、计划总投资963.52亿元。民生福祉有效增进。财政投入民生类支出累计达799.08亿元、占总支出的70.4%，每年坚持办好十件惠民实事，教育、医疗、文化、体育等基本公共服务受益面不断扩大，社保、低保、五保、救助待遇水平逐年提高，农村用水难、行路难、卫生差等短板明显改善，圆满完成两轮扶贫开发“双到”任务。城乡居民人均可支配收入年均增长10%以上，农村居民人均可支配收入超过全省平均水平。生态环境不断改善。环保执法力度持续加大，2016年立案查处数和处罚金额数分别是2011年的1.6倍、2.6倍，近四年城区空气质量优良天数比例提高17个百分点，境内大江大河水质保持优于Ⅱ类标准，西江肇庆段225公里是全国水质最好的河流之一，森林覆盖率提高到70.56%，通过国家环保模范城市、国家卫生城市复检。

　　以上成绩的取得，是省委、省政府和市委正确领导以及市人大、市政协监督支持的结果，是历届各位老领导、老同志鼎力支持和帮助的结果，凝聚着全市人民的智慧和汗水，离不开社会各界的大力支持。我代表市人民政府，向全市人民，向各位人大代表、政协委员，向历届老领导、老同志，向驻肇部队和各级驻肇单位，向所有关心支持参与肇庆建设发展的各界人士，致以崇高的敬意和衷心的感谢！

　　同时，我们也清醒地认识到，全市经济社会发展还存在不少困难和问题。我市刚进入新旧动能转换的初始阶段，长期累积的深层次结构性矛盾还没有得到根本解决，经济发展从较高速增长转入中高速增长周期，GDP增速明显放缓，部分主要经济指标未能完成年度目标任务，人均GDP仍然低于全省、全国同期平均水平，一般公共预算收入增速下滑；产业集聚创新能力不足，高新技术企业数量仅占全省的1%，规上工业企业数量只占全省的2.7%，四大主导产业产值占全市工业比重不足三成，是全省两个没有年产值超百亿元工业企业的地市之一；城市化水平总体偏低，产城融合发展滞后，城镇承载能力不强，房地产去库存压力较大；两大板块发展落差明显，区域发展不平衡问题依然突出，脱贫攻坚任务艰巨；环境难点问题还没有彻底解决，空气质量在全省排名仍然靠后，黑臭水体整治力度有待加大；公共服务供给总量不足、均等化水平不高，民生建设存在不少短板；对行政权力运行的监督制约还不够到位，少数干部不思进取、不敢担当，懒政庸政怠政懈政等“为官不为”现象时有发生，行政服务效能仍需进一步提高，等等。肇庆发展“等不起”、“慢不得”，我们必将采取更加有力的措施解决以上突出问题。

　　回顾过去五年的工作，发展历程令人难忘，发展经验弥足珍贵，我们深刻体会到：第一，必须把加快发展、奋力追赶作为首要任务。发展水平不高仍然是肇庆当前的主要问题，必须切实增强危机感、使命感和紧迫感，坚持发展第一要务，抢抓一切发展机遇，努力实现经济总量和质量的“双提升”、结构和效益的“双优化”，尽快缩小与珠三角核心区的发展差距，加快建设成为名副其实的珠三角城市。第二，必须把振兴实业、壮大产业作为主攻方向。产业是肇庆发展的突出短板，肇庆未来的出路在产业、突破口在产业，必须把产业建设摆在更加重要位置，把招大引强引智作为产业建设的生命线，切实做到亲商扶商、兴业暖企，大力发展实体经济，做大做强产业存量，全力引进产业增量，以产业跨越发展促进经济跨越发展。第三，必须把改革创新、开放合作作为关键动力。抓改革就是抓发展，谋创新就是谋未来，促开放就是增后劲，必须以深化改革破除体制机制障碍，以创新驱动加快转换发展动力，以扩大开放合作增创发展新优势。第四，必须把生态优先、绿色发展作为行动自觉。绿水青山是肇庆的金字招牌，必须像保护眼睛一样保护生态，像对待生命一样对待环境，始终坚持“三个绝不”，毫不放松抓好大气、水、土壤综合整治，推进绿色低碳发展，提升枢纽门户城市宜居宜业宜游形象。第五，必须把以人为本、执政为民作为根本要求。民之所望，政之所向，必须牢记以人民为中心、为民惠民的施政理念，盯住民生短板精准发力，全力办好民生实事，让贫困群众在全面小康建设的路上不掉队，让广大人民群众在共建共享中增强获得感。

　　新一届政府目标任务

　　今后五年，是我市全面建成小康社会的决胜冲刺期，也是我市建设枢纽门户城市的提速攻坚期。当前，我国坚持稳中求进工作总基调，适度扩大总需求，以推进供给侧结构性改革转变发展方式，以深入实施“一带一路”战略扩大开放合作；我省坚定不移推进供给侧结构性改革，大力实施创新驱动战略，着力振兴实体经济，加快建设现代化基础设施，积极推动珠三角与粤东西北产业共建、一体发展，打造粤港澳大湾区，深化泛珠三角和粤桂黔区域合作，拓展大西南和东盟腹地，这些都为我市发展创造了新的重要机遇。我市作为“三大经济带”交汇的重要节点，拥有珠三角创新发展和粤东西北加快发展双重政策叠加支持，成为广东西进、大西南东靠的前沿门户和汇聚东西部经济要素的战略要地，发展空间广阔，发展潜力巨大。站在发展新起点，肇庆市第十二次党代会科学谋划未来五年的宏伟蓝图，确立了“东融西联”的战略取向，为我市发展指明了方向和路径。只要我们增强战略自信，保持发展定力，真抓实干善干，勇于争先进位，就一定能够开创跨越发展的新局面。

　　新一届政府工作总体要求：全面贯彻党的十八大、十八届三中、四中、五中、六中全会和习近平总书记系列重要讲话精神，牢固树立新发展理念，适应把握引领经济发展新常态，坚定不移贯彻稳中求进工作总基调，坚持把供给侧结构性改革作为经济发展的主线，按照省委、省政府和市第十二次党代会各项决策部署，深入实施《珠三角规划纲要》，大力实施“东融西联”战略，推进产业强市，强化创新驱动，深化改革开放，发展实体经济，努力实干兴肇，全力打造珠江—西江经济带和珠江西岸先进制造业重要基地、珠三角城市群联通大西南重要门户、珠三角连接大西南重要交通枢纽，建成国家生态文明示范市，实现工业振兴、产城共兴、城乡齐兴、绿富同兴，全力以赴与全省如期同步率先全面建成小康社会，全面提升珠三角连接大西南枢纽门户地位。

　　新一届政府工作目标任务：未来五年，经济增长速度高于全省平均水平，全面完成“十三五”规划目标任务，生产总值年均增长9%左右，人均生产总值年均增长8%，城乡居民人均可支配收入年均增长9%左右；到2018年，GDP和城乡居民人均可支配收入均比2010年翻一番，与全省同步率先全面建成小康社会；到2021年，GDP达到3200亿元左右，人均GDP达到75000元。

　　围绕总体要求和目标任务，重点推进以下工作：

　　——解放思想对标先进，全力推进“东融西联”。开展新一轮思想大解放，切实增强珠三角意识，树立敢为人先的改革精神、开放包容的宽广胸怀、对标先进的不懈追求、与时俱进的创新意识、亲商扶商的重商理念、实干担当的务实作风，进一步激发干部队伍干事创业热情，提高各级各部门工作效能，对标珠三角核心发达地区，一步一个脚印奋力追赶。坚定不移实施“东融西联”，突出以“东融”作为关键着力点，推动广佛肇同城发展，加快融入珠三角交通快捷圈、产业共建圈、区域协作圈、城市生活圈，尽快实现与珠三角核心区在基础设施、产业共建、高端要素、公共服务等领域全方位深度融合，协同共建珠江西岸先进装备制造产业带；积极以“西联”作为发展助推器，协同共建珠江—西江经济带和粤桂黔高铁经济带，抢占珠三角西进先机，以交通联接构筑枢纽、以产业联手共建基地、以要素联合打造门户，深化与大西南联动合作。加强分类指导、分类施策、一体发展，力促两大板块各具特色竞相发展，加快把东南板块建设成为广佛肇一体化发展、融入珠三角核心区的“排头兵”，努力把西北板块建设成为珠三角联动大西南的“桥头堡”。突出把交通建设作为“东融西联”的先行工程，构筑“十横八纵”交通大动脉，建成内环、外环高速公路和与广佛同标准对接的城际干线路网，构建广佛肇半小时交通圈和山区县连通中心城区一小时交通圈，谋划启动对接广佛的地铁建设，打造综合枢纽站场，加快发展枢纽经济，尽快建成更高水平的珠三角连接大西南综合交通枢纽。

　　——突出抓好产业强市，着力构建现代产业体系。坚持工业优先，立足工业主阵地，做大做强工业经济，切实改变产业发展“小、散、弱、粗”的落后现状。大力实施工业发展“366”工程，培育新能源汽车、先进装备制造、节能环保等3个千亿产业集群，引育6家主营业务收入超百亿元工业企业，新增600家主营业务超亿元工业企业。突出抓好整车制造龙头带动，打造立足珠三角、面向全国的新能源汽车产业基地。以产业共建为抓手，以汽车零部件、智能装备制造为主攻方向，打造具有核心竞争力的先进装备制造产业集群。大力引进战略性新兴产业，着力打造华南地区节能环保产业新高地，扎实推进高端电子信息研发制造基地建设。加快推进新一轮技术改造，促进传统优势产业从点式发展向链式发展转型。构建以肇庆高新区为核，高要汽车零部件产业园、四会产业园为两极，肇庆工业园、肇庆新区产业园、广佛肇经济合作区、粤桂合作特别试验区（肇庆）以及各县（区）产业园为点的“一核两极多点”园区发展新格局，未来五年新增或盘活工业用地超50平方公里，促进形成“一县一核心园区一特色主导产业”，努力打造一批千亿级、百亿级产业园区。力促企业“顶天立地铺天盖地”，全力引进大项目培育大企业，积极扶持发展高新科技、高成长性中小企业。进一步放宽市场准入，全面激发民营经济发展活力，扶持民营企业发展壮大。深入推进农业供给侧结构性改革，加快农业提质增效，推动传统农业大市迈向现代农业强市。促进旅游产业转型升级，加快发展生产性服务业，积极发展港口物流、商贸会展等产业，培育壮大“互联网+”等新经济新业态。做优第一产业，做强第二产业，做精第三产业，构建起特色鲜明、结构优化、竞争力强的现代产业体系。

　　——加快提升城市门户功能，促进产城人融合发展。积极参与粤港澳大湾区和珠三角城市群规划建设，统筹推进“一江两岸五区”大城区同规同建同管，推动肇庆从“环湖城市”走向拥江发展城市，打造成为珠三角世界级城市群中融“山湖城江”为一体、宜居宜业宜游的高品质新都市。强化以人为核心的新型城市化导向，坚持以产促城、以城兴产，优化产城布局衔接，强化智慧城市、低碳城市和海绵城市建设，大力发展城市经济，以产业集聚引导促进人口集聚。推动肇庆新区快速崛起，高水平构建市政基础设施和公共服务体系，强化产业导入，全力打造“五大产业组团”，加快建设国家绿色生态示范城区，确保“八年见新城”。以“两保护、三提升”推动端州老城改造提升，重点实施肇庆府城保护与复兴项目，提升城市品质，延续千年文脉，努力打造成为展现岭南文化魅力的旅游目的地。坚持以城带乡、城乡一体，加快建设县域新城、特色小镇、美丽乡村。

　　——全面激活发展动能，不断增强发展后劲。突出问题导向，深化重点领域和关键环节改革，抓住供给侧结构性改革主线，创新财税、金融和农村产权、土地等制度，深化政府“放管服”改革，进一步简政放权，营造更加有利于发展的制度环境。强化创新驱动引领，力促肇庆高新区打造成为科技创新排头兵，高企数量占园区规上工业企业总量一半以上，在引育百亿企业、千亿产业集群上率先突破，力争5年内跻身国家高新区前70位，成为国内一流的国家高新区；以科技“四众”带“双创”、促“四链深度融合”，加快培育发展新型研发机构，实现高新技术企业五年倍增，争创国家知识产权示范城市；深入实施“西江人才计划”，形成“引进人才—培育企业—催生产业”的链式效应。牢固树立“人人都是招商主体、处处都是投资环境”理念，努力做到审批事项最少、办理速度最快、审批效率最高、行政服务最优。全面提升开放型经济发展水平，加大引资引智引技力度，力争每年引进一批投资超20亿、超50亿乃至100亿项目。坚持实施重大项目带动，着力提升产业项目投资比重。全方位拓展融资渠道，充分发挥金融支持发展的重要作用。

　　——始终坚持绿富同兴，进一步提升人民群众获得感。践行绿色发展理念，坚持“三个绝不”，实行最严格环境保护制度，坚决保护西江母亲河，狠抓环境空气综合防治，推动土壤综合治理，推进新一轮绿化肇庆大行动，共建珠三角国家绿色发展示范区，力争2020年建成国家生态文明示范市。坚持以人为本理念，全面补齐民生短板，推动教育、医疗、社保、文化等公共服务城乡一体化，率先实现教育现代化，医疗卫生综合发展水平达到全省中上水平，构建更加健全的与经济发展同步的社会保障体系，办好第十五届省运会和第八届省残运会，争创全国“双拥模范城”，让广大人民群众享受更加优质的公共服务。拓宽城乡居民收入渠道，实现居民收入倍增，确保到2018年打赢9.7万贫困人口脱贫攻坚战。全面推进依法治市，确保到2018年与珠三角核心区同步率先基本建成法治政府，努力建设珠三角法治创新示范区。

2017年工作安排

　　今年是新一届政府开局之年，全市经济社会发展主要预期目标是：地区生产总值增长7%，人均生产总值增长6.3%，规模以上工业增加值增长8%，固定资产投资增长8%，社会消费品零售总额增长10.5%，实际吸收外资总额增长4%，外贸出口总额增长1%，一般公共预算收入稳定增长，城乡居民人均可支配收入增长与经济增长同步，居民消费价格总指数涨幅控制在3%左右，城镇登记失业率控制在3%以内，人口出生率和自然增长率、节能减排和空气质量改善目标控制在省下达的计划以内。重点抓好以下十个方面的工作：

　　一、推进供给侧结构性改革，多措并举稳增长促发展。继续实施“三去一降一补”行动计划，推进陶瓷产业转型升级，打造陶瓷原材料集中制粉基地，促进陶瓷行业向绿色生产和智能制造方向发展；探索建立租售并举住房制度，推动住房租赁平台全面投入运作，推行保障性住房和棚改货币化安置，全年在消化新增商品房供应的基础上减少14万平方米商品房库存，促进房地产市场持续健康发展；在巩固大幅减负成果基础上，继续打好降成本“组合拳”，落实减税清费政策，力争企业生产要素成本和制度性交易成本低于周边地区。促进“暖企行动”制度化、常态化，帮助企业通过直购电降低用电成本，引导企业用好贷款风险补偿基金和应急转贷资金。抓好企业促建促产和增资扩产，推动更多企业从“四下”转为“四上”，力争全年实现年主营业务收入超10亿元企业达到75家、新增规上工业企业43家。放手发展民营经济，制定加快民营经济发展实施意见，争取更多民营企业列入省重点培育民营骨干企业名录。积极扩大有效投资，构建重大项目建设纵横协调联动管理机制，探索实施项目建设直接落地改革，实行重点产业项目单列管理服务，全年安排市重点项目100项（分解后200个子项目）、计划投资513.2亿元，其中重点产业项目78项、计划投资109.4亿元。加快建设新一代信息基础设施，拓展新能源汽车充电网络。大力发展电商区域总部经济，推动京东集团“一基地三中心”、益华全球商品流通展示体验中心等重点项目尽快建设，促进网上消费效益本地化。以“旅游+”为切入点发展全域旅游，促进文化旅游和城市旅游、乡村旅游、生态旅游融合发展，强化旅游区域合作，推动恒大文化旅游城、金域集团鼎湖健康养生城等重大项目加快建设，打造一批体验式智慧型旅游精品，全力创建星湖国家5A级旅游景区。引进培育一批金融机构，大力发展保险业和创投、风投等股权投资，做大做强金融产业。引导金融机构加大力度支持实体经济发展，切实加强金融风险防范，充分利用绿色金融工具支持环保产业发展，力争新增10家企业上市（挂牌）。积极推进金融改革创新，继续推动农信社改制为农商行，支持银行创新金融业务和金融工具，开展投贷联动融资业务试点，探索实施“股权+债权”融资模式。积极争取政策性银行等金融机构扩大融资，成立城市基础设施建设基金，力争政府性投资项目融资达300亿元。

　　二、打好市政交通建设攻坚战，加快构建外联内通交通网。全年组织实施总投资180亿元的市政交通重点工程41项。加快广佛肇高速广州石井至肇庆大旺段和汕昆、汕湛、怀阳高速肇庆段项目建设，全力推进对接广佛的“两桥一路”项目，动工建设肇开高速，积极推进广肇高速扩建、广茂铁路升级改造，积极谋划柳广铁路、广湛高铁、肇深高铁、肇顺南城际轨道、德罗高速等项目。启动市汽车客运总站搬迁建设，力争明年建成市火车站综合体、肇庆东站客运枢纽。完成西江、北江航道肇庆段扩能升级改造，高起点谋划建设肇庆港中心港区，规划建设肇庆国际多式联运物流园等现代化物流园区，加快西江沿线公共物流码头建设。加快建设肇庆环城快速干线（高要莲塘—金利—四会大沙），基本建成国道321城市化道路改造一期、二期工程并动工建设三期工程，大力推进北岭路网建设以及江滨堤路、端州路、天宁路改造升级，在抓好去年端州城区已开工“瓶颈路”建设的同时，今年再启动建设18条。增强城市公共交通功能，切实缓解交通拥堵。

　　三、全力实施工业发展“366”工程，培育壮大主导产业。制定工业发展“366”工程专项规划和产业发展扶持政策，对纳入“366”工程的重点企业和重点项目实行专门扶持和“一对一”服务。设立产业发展基金，吸引社会资本共建重大科技专项创业投资基金、珠西装备子基金、天使投资子基金，加大对主导产业发展的支持。大力开展“园区建设年”活动，力促产业园区提质增效，突出把肇庆高新区作为实施工业发展“366”工程的主战场，加快肇庆大旺产业转移园扩园发展，抓紧推进鼎湖区申报设立省级产业转移集聚地和端州高新技术产业园、双龙现代产业园规划建设，大力推动广晟集团投资35亿元建设通用厂房和周边基础配套工程，年内新增及盘活园区工业用地10平方公里、新建工业通用厂房100万平方米，产业园区（产业集聚地）实现工业总产值占规上工业比重提高到45%。大力推进与珠三角核心区等发达地区产业共建，积极争取省属国有企业新投资工业项目落户，抓好列入珠江西岸先进装备制造产业带重点台账项目，拓展汽车零部件产业与广佛整车基地合作，加快鸿图科技园和肇庆（高要）智能制造研究院建设，力争完成装备制造业投资193亿元、增加值235亿元；大力推进新能源动力产业小镇、中电新能源汽车等重点项目，迅速壮大新能源汽车整车制造产业集群；加快建设启迪环保科技城、中丹环保产业园，发展节能环保全产业链；力促中国电科肇庆产业园尽快动工，推动电子信息产业向高端环节延伸。优化发展现代服务业，重点培育壮大科技服务、融资租赁、检验检测等生产性服务业，加快建设大数据云服务产业园。

　　四、坚持创新驱动引领，加快转型升级步伐。强化肇庆高新区协同创新引领地位，落实国家自主创新示范区建设方案和“1+N”科技创新政策体系，实施高新技术企业、新型研发机构和发明专利三年倍增计划，加快建设科技金融产业融合创新发展试验区，尽快建成国家级科技企业孵化器。加大科技投入，创新“政银科贷”融资产品，带动企业增加研发资金，力争全市研发投入占GDP比重提高到1.5%。落实高新技术企业和高新技术产品奖补政策，力争新增高新技术企业45家，建成企业孵化器10家，高新技术产品产值占规上工业总产值比重提高2个百分点。抓好产学研用协同创新平台建设，大力引进国家级人才，积极建设院士工作站，加快引进国内外一流高等院校和科研院所建设新型研发机构，加快推进5亿元以上大型工业企业设立研发机构，建设一批科技企业孵化器和中小企业服务平台。打造高要金利五金产业转型升级示范区，加快传统优势产业转型发展。采取智能生产、设备更新、品牌打造、管理创新等手段，继续推进新一轮技术改造，确保50%规上企业实施技改。办好创新创业大赛，引导社会资源参与创新创业。

　　五、狠抓大招商招大商，提升开放型经济发展水平。组织实施“招商引资年”活动，进一步压实招商工作小组和各县（市、区）引资责任，围绕主导产业、大型企业、上市公司精准招商，办好一批主题招商活动和“肇庆金秋”经贸洽谈会，力争全年引进超10亿元项目10个。加大力度吸引外商投资，鼓励支持外资企业增资扩产。建立招商引资统计跟踪台帐，定期核查历次招商签约项目，全面提升项目履约率和资金到位率。完善“三互”大通关改革，打造“南沙港—肇庆码头口岸—园区”物流通道，壮大发展一般贸易，加快加工贸易转型升级，积极发展服务贸易和服务外包，促进跨境电子商务发展，规范发展旅游购物贸易。积极对接珠江西岸先进装备制造产业带、珠江—西江经济带、粤桂黔高铁经济带沿线城市开展产业互补共建，谋划建设粤桂黔高铁经济带合作试验区广东分园肇庆园（怀集）。深化广佛肇经济圈合作，全力争取广州国际汽车零部件产业基地项目落户，辐射带动肇庆（高要）汽车零部件产业园发展；加快广佛肇经济合作区“一主两辅”产业发展，新增开发面积2000亩、引进项目20个。积极向省申报实施粤桂合作特别试验区（肇庆）“1+3”配套方案，推动粤桂叠加支持政策落地，建成使用粤桂公共服务中心，大力开展联合招商，加快示范园基础设施建设，共建两广金融改革创新综合试验区。

　　六、统筹抓好新区建设和老城复兴，加快城市扩容提质。构建完善“一张蓝图干到底”工作机制和专项规划协调机制，形成全市“多规合一”的“一张底图”规划平台和“四类控制线”管控体系。加快肇庆新区起步区建设，力争新获批政府性融资150亿元，抓紧推进地下综合管廊、四大水系、肇庆东站站前综合体等在建重点项目，加快肇庆东站至321国道连接线、长利大道、新港路、站前大道等主次干道建设，建成“两馆一宫”和新区中学，力争引进“五大产业组团”项目10个。加快实施肇庆府城保护与复兴项目，大力推进古城墙修缮保护、两广总督府重建、包公府衙修复等重点项目，年内完成市博物馆新馆主体工程。统筹“一江两岸五区”大城区一体化规划建设，促进端州城区产城互动，提升城市功能；推动鼎湖区打造若干个高品质城市功能组团，加快与新区对接融合；确保建成阅江大桥，加快肇庆大桥改造扩建，继续谋划发展跨江交通，打造“一江两岸”景观带，促进高要与端州融合发展。开展智慧城市和海绵城市建设试点，推进城市管理执法体制改革，设立市、县两级城市综合管理机构和数字化管理平台。加大土地管理力度，抓好省节约集约用地试点示范建设工程，全年盘活闲置土地约3000亩，实施“三旧”改造约1500亩，利用旧厂房谋划建设若干个文化创意园。

　　七、做大做强县域经济，扎实推进“三农”工作。全面落实省进一步加快县域经济社会发展的决定，以“五化”促进县域特色差异化发展。坚持产业强县、特色兴县，加快建设一批新型工业化产业示范基地，积极承接产业链集群式组团式转移，争取珠三角发达地区龙头企业布点县域建设生产基地，培育发展特色支柱产业，打造一批产业升级示范镇和专业镇。完善以县城为中心的县域城镇体系，全面放开县城和建制镇落户限制，开展编制省级中心镇控制性详细规划试点，加快培育建设省级特色小镇，保护历史文化名村和传统村落。提高县域高速公路密度，尽快实现乡镇基本通省道。推进农田水利基础设施建设，新建高标准农田18.65万亩。以供给侧结构性改革调整优化农业结构，壮大发展现代农业，打造一批绿色优质农产品品牌，扶持发展南药、珍贵树种种植等特色优势农林产业。抓好广东（高要）粤台农业合作试验区和肇庆（鼎湖沙浦）现代农业科技示范园建设，开展农民合作社、家庭农场示范创建活动，发展农业规模经营。利用“互联网+现代农业”打造农产品电商产业，发展农产品冷链仓储物流。深化农村综合改革，稳步推进农村土地所有权、承包权、经营权三权分置，完成农村土地承包经营权确权登记颁证，推动农村普惠金融服务“村村通”。落实建设美丽乡村“三年行动计划”，全力推动村庄规划全覆盖，整县连片推进农村人居环境“五项整治”，新增省卫生村受益人口8万人以上，确保到2018年自然村基本完成人居环境综合整治任务。完成农村道路硬底化300公里，尽快实现村村通自来水，加快新一轮农村电网改造，实现所有20户以上自然村通光纤。压实对口帮扶责任，抓好产业扶贫、金融扶贫、教育扶贫、就业扶贫、低保扶贫，构建造血式扶贫长效机制，力争相对贫困户农民人均纯收入增幅高于全省平均水平，确保全年实现4万贫困人口脱贫。

　　八、奋力创建全国文明城市，建设文明绿色生态美丽家园。对照全国文明城市创建标准，加大力度开展“8+1”专项整治，进一步提升市民文明素质和城市文明程度，确保创建成为全国文明城市。深入挖掘特色文化资源，培育旅游文化创意产品，建立文化产业融资、产品交易、公共服务平台，加快市文化发展中心、崇禧塔中西文化园建设，免费向公众开放公共文化场馆，继续开展“周末剧场”、“百姓大舞台”等群众文化活动。深入开展“美丽肇庆·幸福家园”和文明镇街系列活动，广泛开展“肇庆暖”等公益性志愿服务，创建宜居社区25个、宜居城镇10个。加快建设环保基础设施，开展新一轮生活垃圾和污水处理设施建设，启动市生活垃圾无害化处理场扩容（三期）工程，城镇生活垃圾无害化处理率提高到90%，启动建设污水处理厂25座，城镇污水处理率提高到87%以上。建设天然气管网50公里，天然气气化率提高到30%。继续开展“环境执法年”活动，探索建立更严格的环境保护制度，做好环境监察监测执法垂直管理改革，落实中央环保督察交办案件整改工作，解决一批环保突出问题。强化大气污染“五大重点领域”综合治理，确保城区优良天数比例稳步上升。保持打击盗采河砂高压态势，落实西江水质保护负面清单，积极开展中小河流和小流域治理，以“河（涌）长制”强化“一河一策”河涌治理，确保西江、绥江水质安全，饮用水源地水质100%达标。摸清土壤污染底数，开展土壤污染治理修复试点。深入实施新一轮绿化肇庆大行动，加快推进林分改造，新建森林公园22个、湿地公园4个、乡村绿化美化工程174个，完善提升高速公路景观林带100公里。强化节能减排降碳约束性指标管理，实施工业能效赶超行动，加快推广清洁能源利用、建筑节能、新能源汽车。

　　九、加快公共服务均等化，让发展成果更多惠及人民群众。加快补齐率先全面建成小康社会民生短板，力争年内基本完成主要任务。强化农村转移劳动力、城乡就业困难人员、退役军人等特定人群专项职业技能培训，加大对大学生、返乡青年和妇女创新创业支持，加快建设综合性创业孵化（实训）基地，新增城镇就业4.15万人、城镇失业人员再就业1.15万人。加快教育现代化，促进教育优质均衡发展，推进义务教育学校标准化建设，提高高中阶段教育质量，发展公益普惠性学前教育，解决城镇优质学位不足和大班额问题。实施乡村教师支持计划，建立乡村教师荣誉制度，全面落实乡村教师生活补助政策。支持肇庆学院省市共建应用型本科院校、肇庆医专和广东工商学院“升本”。推动职业教育优质化、专业化、集团化发展，积极创建现代职业教育综合改革示范市。扎实推进卫生强市建设，建设高水平区域医疗卫生中心，开展医疗卫生强基创优行动。加快实施市第三人民医院新住院大楼等市县重点医疗卫生项目，完成乡镇卫生院标准化建设，继续推进公建民营村卫生站规范化建设。统筹实施医保支付、分级诊疗、基本药物等政策，深化县级公立医院综合改革，鼓励社会力量举办各类医疗机构。建设市级区域、基层医疗卫生机构信息平台系统，探索“互联网+医疗”健康服务体系。切实抓好传染病防控。落实计划生育目标管理责任制。健全社会保障体系，提高退休人员基本养老金标准，加快医保支付方式改革，完善城乡居民大病保险和医疗救助制度，城乡居民基本医疗保险参保率稳定保持98%以上、年人均政府补助标准不低于420元，城镇、农村低保人均补差水平分别提高到每人每月457元和206元。全年开工建设棚户区改造住房2823户，建成保障性住房、棚户区改造住房3202套（户），完成省下达的农村危房改造任务，抓紧解决乡镇教师和医务人员等群体住房困难。加快公共体育服务均等化，集中力量推进市体育中心、肇庆新区体育中心等省运会场馆建设改造。强化食品药品安全监管，积极创建省质量强市示范城市。继续推进怀集县下帅乡创建全省民族发展先进乡。铁腕整治安全生产隐患，构建重预防、抓治本的安全生产长效机制。推进公共法律服务均等化，健全预防与依法化解社会矛盾纠纷机制，整合贯通“八张防控网”，坚决打击违法犯罪，深化“平安肇庆”建设。继续做好统计、民族、宗教、革命老区、外事、侨务、港澳、对台、人防、国防动员、地方志、档案、气象、水文、地震、妇女儿童、老龄、残疾人、慈善等工作。在办好省十件民生实事的同时，继续集中力量办好市十件惠民实事（具体内容见附件一）。

　　十、强化政府作风建设，努力打造法治高效廉洁政府。全面贯彻党的十八届六中全会、省委十一届八次全会和市第十二次党代会精神，把政府自身建设摆在突出重要的位置。严格依法行政，全面落实我市《法治政府建设实施纲要》，依法定期向人大及其常委会报告工作和向政协通报情况，办好人大建议和政协提案，落实重大行政决策公众参与、专家论证、风险评估、合法性审查、集体讨论和实施后评估制度，实施重大行政决策目录管理制度，完善行政裁量权基准制度，基本建立行政执法信息平台。推进高效施政，加大“放管服”改革力度，实施政府部门权责清单动态管理，严控新设行政审批事项，出台市本级社会组织购买服务资质目录，抓好高要区金利镇简政强镇改革试点；开展“减证便民”专项行动，全面清理精简各类证明手续，推进商事登记后置行政审批事项分类改革，大幅削减商事登记前置和后置审批事项，深化企业“五证合一、一照一码”改革，提高企业开办便利度；完善“一门式一网式”政府服务，实现市级部门、条件允许的省垂直管理部门的审批系统与省网上办事大厅肇庆分厅全面对接，行政审批事项网上全流程办理率和办结率分别达到80%、70%以上。强化廉洁从政，落实政府系统全面从严治党主体责任，加强对行政权力运行监督制约，加大力度惩治腐败，严格审计监督、财政专项资金和“三公”费用监管，持之以恒纠正“四风”，铁腕整治不作为、慢作为、乱作为的庸懒懈怠现象，落实容错纠错预警机制，构建“亲”“清”互动新型政商关系，营造风清气正的政务环境。

　　各位代表！建设枢纽门户城市，全面建成小康社会，是历史的使命、时代的呼唤和全市人民的期盼。让我们紧密团结在以习近平同志为核心的党中央周围，在省委、省政府和市委的正确领导下，认真贯彻落实市第十二次党代会精神，大力实施“东融西联”战略，同心同德，坚定信心，砥砺奋进，以优异的成绩迎接党的十九大胜利召开，为与全省同步率先全面建成小康社会、加快建设珠三角连接大西南枢纽门户城市而努力奋斗！
